# Secretion of Gluconic Acid From *Nguyenibacter* sp. L1 Is Responsible for Solubilization of Aluminum Phosphate

**DOI:** 10.3389/fmicb.2021.784025

**Published:** 2021-11-18

**Authors:** Xiao Li Li, Xue Qiang Zhao, Xiao Ying Dong, Jian Feng Ma, Ren Fang Shen

**Affiliations:** ^1^State Key Laboratory of Soil and Sustainable Agriculture, Institute of Soil Science, Chinese Academy of Sciences, Nanjing, China; ^2^University of Chinese Academy of Sciences, Beijing, China; ^3^Institute of Plant Science and Resources, Okayama University, Kurashiki, Japan

**Keywords:** *Nguyenibacter*, *Lespedeza*, aluminum phosphate, gluconic acid, iron phosphate, aluminum toxicity, carbon source

## Abstract

Phosphorus (P) deficiency is one of the major factors limiting plant growth in acid soils, where most P is fixed by toxic aluminum (Al). Phosphate-solubilizing bacteria (PSBs) are important for the solubilization of fixed P in soils. Many PSBs have been isolated from neutral and calcareous soils, where calcium phosphate is the main P form, whereas PSBs in acid soils have received relatively little attention. In this study, we isolated a PSB strain from the rhizosphere of *Lespedeza bicolor*, a plant well adapted to acid soils. On the basis of its 16S rRNA gene sequence, this strain was identified as a *Nguyenibacter* species and named L1. After incubation of *Nguyenibacter* sp. L1 for 48 h in a culture medium containing AlPO_4_ as the sole P source, the concentration of available P increased from 10 to 225 mg L^–1^, and the pH decreased from 5.5 to 2.5. *Nguyenibacter* sp. L1 exhibited poor FePO_4_ solubilization ability. When the pH of non-PSB-inoculated medium was manually adjusted from 5.5 to 2.5, the concentration of available P only increased from 6 to 65 mg L^–1^, which indicates that growth medium acidification was not the main contributor to the solubilization of AlPO_4_ by *Nguyenibacter* sp. L1. In the presence of glucose, but not fructose, *Nguyenibacter* sp. L1 released large amounts of gluconic acid to solubilize AlPO_4_. Furthermore, external addition of gluconic acid enhanced AlPO_4_ solubilization and reduced Al toxicity to plants. We conclude that secretion of gluconic acid by *Nguyenibacter* sp. L1, which is dependent on glucose supply, is responsible for AlPO_4_ solubilization as well as the alleviation of Al phytotoxicity by this bacterial strain.

## Introduction

Phosphorus (P) is an essential macronutrient required for plant growth and productivity (Tania et al., 2010). Even in P-rich soils, more than 80% of P is immobile and not readily accessible for plant uptake (Xu et al., 2020). P exists in soil in different forms, mainly as inorganic and organic P, whose proportions are soil dependent (Cross and Schlesinger, 1995). The forms of inorganic P also vary in soil as the function of soil pH (Bashan et al., 2013). In alkaline soils, inorganic P exists mainly in the form of calcium phosphate, magnesium phosphate, and octacalcium phosphate. In acid soils with high level of weathering, iron (Fe) and aluminum (Al) oxides strongly absorb P to form fixed Fe-P and Al-P (Hemwall, 1957; Tian et al., 2021). At present, approximately 40% of cultivated land worldwide comprises acid soils (von Uexküll and Mutert, 1995). In China, the area occupied by acid soils is 2.18 million km^2^, which covers 22.7% of the total area of the country (Huang and Zhao, 2014). In the acid soil region of southern China, P deficiency has become a limiting factor in crop production (Zhao et al., 2014; Wang et al., 2021). Although large amounts of P fertilizers are applied to soil to sustain high crop production, less than 20% of added P is used by crops, and most of it is fixed in soil (Podile and Kishore, 2007). Enhancing plant utilization of immobilized P in soil is therefore an important strategy for decreasing the application of P fertilizer.

Two approaches can be used to improve plant utilization of fixed P in soil (Shen et al., 2011). One method involves improving the efficiency of P utilization by plants themselves—for example, by generating P-efficient plants (Fageria et al., 1988; Swamy et al., 2019); the other is increasing the bioavailability of insoluble P by improving soil, such as by inoculating or enhancing phosphate-solubilizing bacteria (PSBs) in soil (Sarkar et al., 2012; Awais et al., 2017). Given the increasing costs of chemical fertilizers and their negative environmental impacts, the application of PSBs is a promising contribution to the development of sustainable agriculture (Etesami et al., 2021).

Rhizosphere processes are important for plant P acquisition (Shen et al., 2011). PSBs participate in a series of rhizosphere processes affecting the transformation of soil P, especially the solubilization and mineralization of insoluble P, thereby improving the P acquisition capability of plants. Many screening studies have been carried out to identify PSB strains with high solubility for tricalcium phosphate in neutral to alkaline soils, including members of *Bacillus*, *Pseudomonas*, and *Stenotrophomonas* (Chen et al., 2006; Wahid et al., 2020), and *Agrobacterium*, *Acinetobacter*, *Pantoea*, and *Burkholderia* (Lin et al., 2006; Zineb et al., 2019). Some of these strains have been applied in field experiments with remarkable results. For example, a phosphate-solubilizing *Bacillus* sp. significantly enhanced seed cotton yield and plant height (Qureshi et al., 2012). In another study, the PSB strain *Klebsiella variicola* in combination with arbuscular mycorrhizal fungi strain *Rhizophagus intraradices* promoted plant growth under field conditions (Nacoon et al., 2021). Most of these PSBs, however, exhibited much lower inorganic P solubilization (only 0.16%) in acid soils, where Al-P and Fe-P are the dominant forms (Zhang et al., 2020). Screening of indigenous PSBs to identify those with high abilities to solubilize Al-P and Fe-P in acid soils is therefore of great value.

*Lespedeza bicolor* is a leguminous shrub that grows well in infertile acid soils (Chen et al., 2010). This species can survive in soil at a pH of 4.5 (Vogel, 1981; Cline and Senwo, 1994) and has been found to still be very productive at pH levels of 4.40 and 5.44 (Hyland, 1938). Al toxicity and P deficiency are two main factors limiting plant growth in acid soils (Zheng, 2010; Zhao et al., 2014). In a previous study, we determined that *L. bicolor* roots secrete a large amount of malate and citrate to neutralize Al toxicity in low-P acid soil (Dong et al., 2008). Still unknown, however, is whether native bacteria in the rhizosphere of *L. bicolor* play a role in its Al tolerance and P acquisition. In the present study, we isolated an aluminum phosphate-solubilizing bacterium from the rhizosphere soil of *L. bicolor* grown in P-limited acid soil and functionally characterized this strain in terms of its solubilization of Al-P.

## Materials and Methods

### Soil Sampling Sites

To isolate Al-P-solubilizing bacteria, we sampled the rhizosphere soil of *L. bicolor* grown in acid soil located at the Yingtan Red Soil Ecological Experimental Station (28°14′N, 117°03′E), China. This area is characterized by a typical subtropical humid monsoon climate with a mean annual precipitation and temperature of 1,882 mm and 18.4°C. The soil is derived from Quaternary red clay and is classified as a Ferric Acrisols (FAO soil classification system). Soil adhering to the roots of *L. bicolor* was shaken off and placed in plastic bags. The soil samples were stored at 4°C. To measure rhizosphere soil pH, we used a pH meter (Mettler Toledo FE20, Shanghai, China) to analyze a soil–water suspension (soil: water, 1: 2.5) after shaking (Zheng et al., 2019). Available P was extracted by the ammonium fluoride method and analyzed by the molybdate blue method (Murphy and Riley, 1962). Soil organic matter was determined by low-temperature external-heat potassium dichromate oxidation-photo-colorimetry (Zhang et al., 2019). Soil texture (clay, silt and sand contents) were measured by Laser Particle Sizer (LS13320, Beckman Coulter Inc., California, United States). Different forms of Fe and Al oxides in soil was extracted according to the following methods: free crystalline Fe and Al oxides (Fed, Ald) were extracted by dithionite-citrate-bicarbonate; amorphous Fe and Al oxides (Feo, Alo) were extracted by acid ammonium oxalate; and complex Fe and Al oxides (Fep, Alp) were extracted by sodium pyrophosphate (Lu, 1998). Al and Fe in the extract solutions were determined by inductively coupled plasma–atomic emission spectrophotometry (ICP-AES; Optima 8000, PerkinElmer, Waltham, MA, United States). The basic properties of the sampled soil were as follows: pH, 4.12; available P, 8.65 mg kg^–1^; organic matter, 14.42 g kg^–1^; clay content, 54.3%; silt content, 28.8%; sand content, 16.9%; Fed, 37.02 g kg^–1^; Ald, 3.57 g kg^–1^; Feo, 0.04 g kg^–1^; Alo, 0.81 g kg^–1^; Fep, 0.03 g kg^–1^; and Alp, 0.74 g kg^–1^.

### Isolation of Aluminum Phosphate-Solubilizing Bacteria

The screening medium consisted of 10 g of glucose, 5 g of MgCl_2_⋅6H_2_O, 0.25 g of MgSO_4_⋅7H_2_O, 0.2 g of KCl, 0.1 g of (NH_4_)_2_SO_4_, 15 g of agar powder, and 1.0 g of AlPO_4_ as an insoluble P source in 1 L of distilled water (pH 5.5). AlPO_4_ and the other components were autoclaved separately and then aseptically mixed (Park et al., 2011). Approximately 2 g of rhizosphere soil collected as described above was aseptically transferred to a conical flask with 50 mL of sterile water and shaken at 180 rpm for 30 min. Afterward, a series of 10-fold dilutions of this suspension was carried out for each sample, and 50 μL of each dilution was plated on the screening solid-culture medium. PSBs were identified by the presence of a clear halo around colonies after 7 days of incubation in the dark at 30°C.

### Genomic DNA Isolation, 16S rRNA Gene Sequencing, and Phylogenetic Analysis

Genomic DNA was isolated using a bacterial DNA extraction kit (Tiangen Biotech, Beijing, China). The DNA samples were sequenced by Genscript Biotechnology Company (Nanjing, China). The 16S rRNA gene of the isolate was amplified by PCR using universal bacterial primers F27 (5′AGAGTTTGATCCTGGCTGGCTCAG-3′) and R1492 (5′-TACGGCTACCTTGTTACGACTT-3′) (Wang et al., 2020). PCR amplification was performed according to the following protocol: hot start at 94°C for 5 min, followed by 30 cycles of denaturation at 94°C for 1 min, annealing at 55°C for 1 min, and extension at 72°C for 2 min, with a final extension of 72°C for 10 min. The 16S rRNA gene sequence of the screened strain reported in this study was submitted to GenBank (accession no. MW774243). The obtained 16S rRNA gene sequence was compared with available standard sequences of bacterial lineages in GenBank using BLAST. A phylogenetic tree was constructed by the neighbor-joining method from distance matrices using MEGA (Thompson et al., 1997).

### Comparison of Al-P and Fe-P Solubilizing Abilities of the Isolated Strain

A 50-μL aliquot of the isolated PSB (OD_600_ = 0.5) was inoculated into 50 mL of liquid culture medium containing 0.05 g of Al-P (AlPO_4_) or Fe-P (FePO_4_). If required, an equal volume of sterile water (CK) or *Escherichia coli* was added as a control. The PSB isolate was cultured on a rotary shaker (180 rpm) in the dark at 30°C. After 0, 2, 4, 6, 8, or 10 days, the amount of soluble phosphate in the culture solution was measured by the molybdate blue method, and the pH of the medium was recorded with a pH meter equipped with a glass electrode.

### Effects of pH, Organic Acid, and Carbon Source on Al-P Solubilization

A 50-mL portion of aluminum phosphate medium was inoculated with PSB isolate as described above. Sterile water added to the medium was treated as a blank. After culture on a rotary shaker (180 rpm) in the dark at 30°C for 0, 12, 24, 36, or 48 h, the pH and soluble P and organic acid contents of the growth medium were determined. pH and soluble P were measured as described above. For the determination of organic acids, bacterial broth was filtrated through a 0.22-μm filter, and 20 μL of each filtrate was subjected to HPLC (UltiMate 3000, Thermo Fisher Scientific, Chelmsford, MA, United States). The separation of organic acids was carried out on a Syncronis C18 column (30°C), with 0.05 M KH_2_PO_4_ in purified water (pH = 2.68) used as the mobile phase. The flow rate was 0.5 mL min^–1^, and the retention time of each signal was recorded at a wavelength of 210 nm.

To explore the effects of pH and gluconic acid on the solubilization of Al-P, 50 mL of aluminum phosphate medium lacking PSB isolate in the presence and absence of 15 g L^–1^ gluconic acid was pH adjusted from 5.5 to 2.5 with 0.1 M HCl. The soluble P concentration in each culture solution was then measured. To explore the effects of carbon source on Al-P solubilization, 50 mL of aluminum phosphate medium supplied with glucose or fructose was cultured with or without inoculation with PSB isolate. After culture on a rotary shaker (180 rpm) in the dark at 30°C for 48 h, the soluble P content and gluconic acid of the growth medium were determined as described above.

### Effect of Gluconic Acid on Al Toxicity to Plants

The Al-sensitive rice cultivar Kasalath (Ma et al., 2002) was used to test the effect of gluconic acid on Al toxicity to plants. The rice seeds were soaked in water at 37°C for 24 h and then placed on a net floating on a solution of 0.5 mM CaCl_2_ (pH 4.5) in a 2-L plastic container. After 2 days, the seedlings were exposed to a solution of 0.5 mM CaCl_2_ (pH 4.5) containing 0, 0.05, 0.5, 2.5, or 7.6 mM gluconic acid with or without 50 μM Al for 24 h. Before and after the 24-h exposure, root lengths were measured with a ruler. All experiments were conducted in an incubator with a relative humidity of 65% at 26°C in the dark. Al was supplied as AlCl_3_⋅6H_2_O.

### Statistical Analysis

Analyzed data consisted of 10 replicates in the root elongation experiment and three replicates in other experiments. Data analysis was performed using Excel software (Excel for Windows 2016, Redmond, WA, United States).

## Results

### The Phosphate-Solubilizing Bacteria *Nguyenibacter* sp. L1 Was Isolated and Identified

**FIGURE 1:**
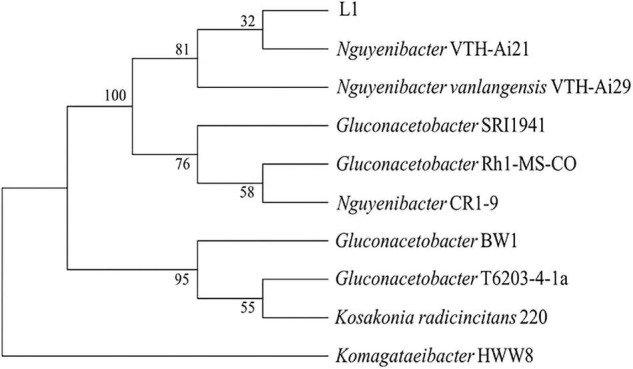
Neighbor-joining phylogenetic tree based on 16S rRNA gene sequences of the screened strain and other bacterial strains. Numbers at nodes are bootstrap values (%).

The efficient Al-P solubilizing bacterial strain L1 was isolated from the rhizosphere soil of *L. bicolor* using culture medium containing AlPO_4_ as the sole P source. Sequence comparison revealed that the 16S rRNA gene sequence of isolated strain L1 was more than 99% identical to those of *Nguyenibacter* sp. VTH-Ai21 (AB971698.1) and *Nguyenibacter vanlangensis* VTH-Ai29 (LC103268.1) (Figure 1). The isolated strain, which was designated as *Nguyenibacter* sp. L1 in this study, was deposited in the China Center for Type Culture Collection (CCTCC no. M2021392).

### *Nguyenibacter* sp. L1 Solubilized Al-P but Not Fe-P

**FIGURE 2:**
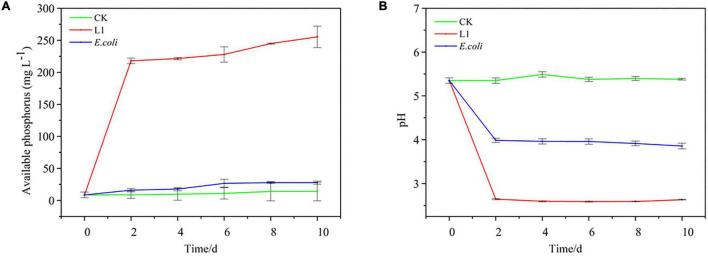
Aluminum-phosphate solubilizing abilities of *Nguyenibacter* sp. L1. **(A,B)** Available P concentration **(A)** and pH **(B)** of the culture medium after culturing *Nguyenibacter* sp. L1 with AlPO_4_ as the P source at 30°C for 0, 2, 4, 6, 8, and 10 days. An *Escherichia coli* strain and sterile water (CK) were included as controls. Vertical bars represent standard deviations of the means (*n* = 3).

**FIGURE 3:**
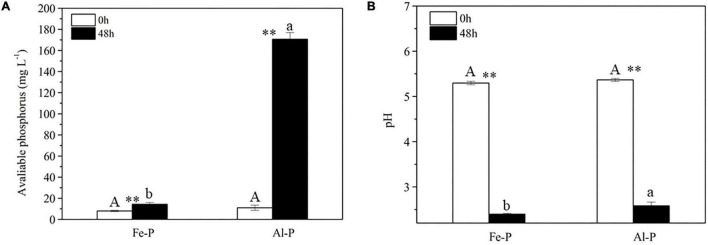
Comparison of Al-P and Fe-P solubilizing abilities of *Nguyenibacter* sp. L1. **(A,B)** Available P concentration **(A)** and pH **(B)** of the culture medium after culturing *Nguyenibacter* sp. L1 with AlPO_4_ or FePO_4_ as the P source at 30°C for 48 h. Vertical bars represent standard deviations of the means (*n* = 3). Different capital and lowercase letters above columns indicate significant differences (*p* < 0.05) between two P sources after culture for 0 and 48 h, respectively. Asterisks above columns indicate significant differences (*p* < 0.01) between 0 and 48 h.

Compared with CK and *E. coli* controls, inoculation with *Nguyenibacter* sp. L1 markedly increased the available P concentration of Al-P-containing culture medium and decreased the pH of the medium after 2 days (Figure 2). Although inoculation with *Nguyenibacter* sp. L1 also significantly decreased the pH of Fe-P-containing culture medium after 2 days, it had little effect on the concentration of available P (Figure 3).

### Growth Medium Acidification and Gluconic Acid Secretion Were Responsible for Al-P Solubilization by *Nguyenibacter* sp. L1

**FIGURE 4:**
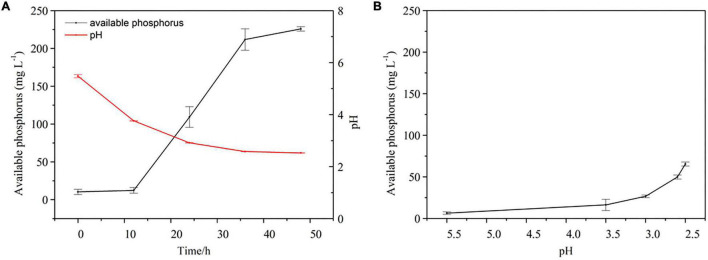
Effect of pH on Al-P solubilization. **(A)** Available phosphorus concentration and pH of the culture medium after culturing *Nguyenibacter* sp. L1 with AlPO_4_ as the P source at 30°C for 0, 12, 24, 36, and 48 h. **(B)** Available P concentration of the culture medium with AlPO_4_ as the P source after manually adjusting the pH from 5.5 to 2.5 without inoculation with *Nguyenibacter* sp. L1. Vertical bars represent standard deviations of the means (*n* = 3).

A time-course experiment over 2 days further confirmed that the increase in the concentration of available P in culture medium containing Al-P was accompanied by a decrease in pH (Figure 4A). To investigate the effect of pH on Al-P solubility, the pH of the culture medium containing Al-P was artificially adjusted from 5.5 to 2.5 without inoculation with *Nguyenibacter* sp. L1 (Figure 4B). Under this condition, the concentration of available P in the culture medium only increased from 6.48 to 65.53 mg L^–1^. This increase due to artificial pH adjustment was much lower than that in medium inoculated with *Nguyenibacter* sp. L1 (Figure 4A). These results suggest that pH reduction is one of the mechanisms responsible for the Al-P solubilizing ability of *Nguyenibacter* sp. L1, although other mechanisms, such as organic acid secretion, may be also involved in the solubilization of Al-P by *Nguyenibacter* sp. L1.

**FIGURE 5:**
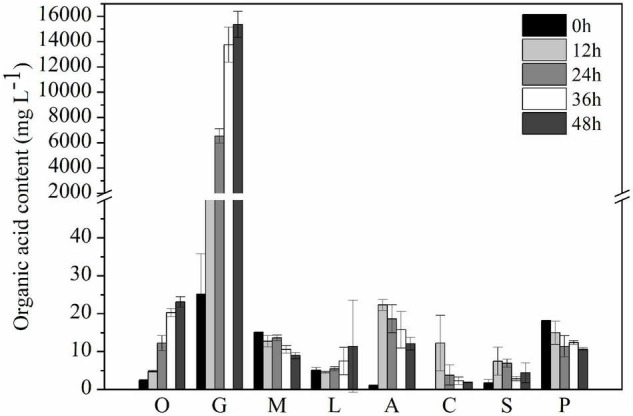
Concentration of organic acids in the culture medium. Secreted organic acids were measured after culturing *Nguyenibacter* sp. L1 with AlPO_4_ as the P source at 30°C for 0, 12, 24, 36, and 48 h. Vertical bars represent standard deviations of the means (*n* = 3). O, oxalic acid; G, gluconic acid; M, malic acid; L, lactic acid; A, acetic acid; C, citric acid; S, succinic acid; P, propionic acid.

**FIGURE 6:**
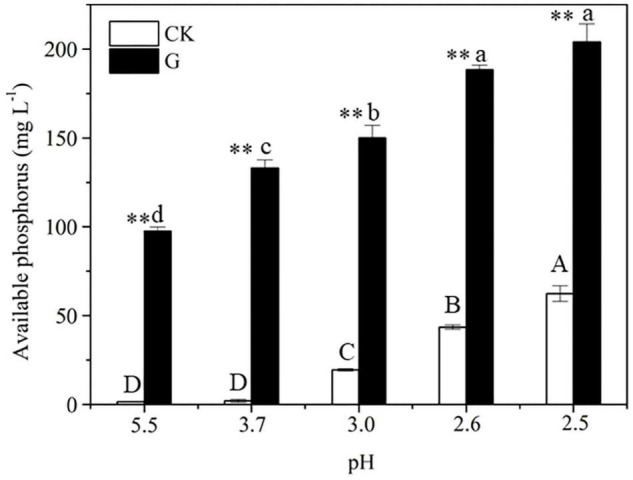
Effect of gluconic acid on Al-P solubilization. The concentration of available P in uninoculated culture medium containing AlPO_4_ as the P source was determined after addition of 15 g L^–1^ gluconic acid at pH 5.5, 3.7, 3.0, 2.6, and 2.5. The pH of the culture medium was manually adjusted as in Figure 4A at 0, 12, 24, 36, and 48 h. Vertical bars represent standard deviations of the means (*n* = 3). Different capital and lowercase letters above columns indicate significant differences (*p* < 0.05) among different pHs without and with gluconic acid, respectively. Asterisks above columns indicate significant differences (*p* < 0.01) between CK and gluconic acid.

**FIGURE 7:**
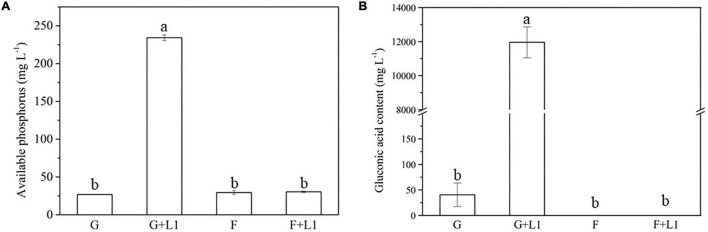
Effects of carbon source on Al-P solubilization and gluconic acid secretion. **(A,B)** Concentrations of available P **(A)** and gluconic acid **(B)** in culture medium containing different carbon sources. Al-P-containing medium with or without added *Nguyenibacter* sp. L1 was cultured at 30°C for 48 h in the presence of glucose (G) or fructose (F), and the concentrations of available P and gluconic acid were determined. Vertical bars represent standard deviations of the means (*n* = 3). Different lowercase letters above columns indicate significant differences (*p* < 0.05) among treatments.

After 2-day cultivation, *Nguyenibacter* sp. L1 produced a variety of organic acids in the culture medium; among them, gluconic acid had the highest concentration, up to approximately 15 g L^–1^ (Figure 5). Furthermore, the concentration of available P was increased by the manual addition of gluconic acid to culture media at different pHs and lacking *Nguyenibacter* sp. L1 (Figure 6). A large amount of gluconic acid and a higher available P concentration were observed in culture medium containing glucose as the carbon source and inoculated with *Nguyenibacter* sp. L1 (Figure 7). When medium containing fructose as the carbon source was inoculated with *Nguyenibacter* sp. L1, however, gluconic acid was not detected in the culture medium, and the concentration of available P was unchanged even though the pH dropped to 3.5. These results suggest that secretion of gluconic acid was responsible for the solubilization of Al-P by *Nguyenibacter* sp. L1, a process dependent on the supply of glucose in the growth medium.

### Gluconic Acid Alleviated Al Toxicity to Plants

**FIGURE 8:**
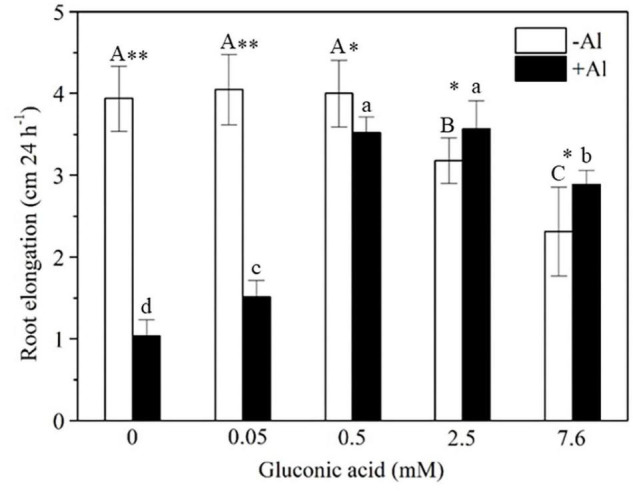
Effect of gluconic acid on Al-induced inhibition of root elongation in rice. Two-day-old rice seedlings (cv. Kasalath) were exposed to a solution of 0.5 mM CaCl_2_ (pH 4.5) containing 0, 0.05, 0.5, 2.5, or 7.6 mM gluconic acid in the absence (–Al) and presence (+Al) of 50 μM Al for 24 h. Root length was measured with a ruler before and after treatment. Vertical bars represent standard deviations of the means (*n* = 10). Different capital and lowercase letters above columns indicate significant differences (*p* < 0.05) among different concentrations of gluconic acid under –Al and + Al, respectively. Asterisks above columns indicate significant differences (^∗^*p* < 0.05; ^∗∗^*p* < 0.01) between –Al and +Al.

To examine whether gluconic acid is able to detoxify Al, we compared the inhibitory effect of Al on the root elongation of rice plants in the presence or absence of gluconic acid. Rice root elongation was markedly inhibited by 50 μM Al with or without 0.05 mM gluconic acid, but this inhibitory effect disappeared in the presence of 0.5, 2.5, or 7.6 mM gluconic acid (Figure 8). This result indicates that the external addition of gluconic acid alleviated Al toxicity to plant roots.

## Discussion

In this study, we isolated a PSB strain with Al-P solubilizing activity from the rhizosphere soil of *L. bicolor* grown in acid soils. On the basis of 16S rRNA gene sequence comparison, the screened strain was found to belong to *Nguyenibacter* and was designated as *Nguyenibacter* sp. L1. *Nguyenibacter* has been previously reported to be a N-fixing bacterium (Thi Lan Vu et al., 2013). Our study has revealed a new function of *Nguyenibacter*, namely, solubilization of Al-P. In contrast, *Nguyenibacter* sp. L1 has little ability to solubilize Fe-P. The solubility product (pKsp) value of Al-P and Fe-P ranges from 28 to 32 and from 33 to 35, respectively, thus indicating the lower solubility of the latter compound (Chang and Jackson, 1957). Some fungi have different abilities to solubilize insoluble phosphates (in the order of Ca-P > Al-P > Fe-P), and the acidity generated by these fungi is consistent with their ability to solubilize Ca-P and Al-P but not Fe-P (Spagnoletti et al., 2016). The PSB strain isolated in this study may therefore similarly be unable to solubilize Fe-P because of the low solubility of Fe-P.

Bacterial solubilization of P is a complicated process. The ability of bacteria to solubilize P is related to their growth conditions, such as nutritional, physiological, and growth aspects of the culture (Reyes et al., 1999). Acidification of the medium can facilitate the solubilization of fixed P forms (Illmer and Schinner, 1995; Park et al., 2011). In the present study, inoculation with *Nguyenibacter* sp. L1 decreased the pH of the growth medium, which also led to an increase in available P. In addition, organic acids can compete with phosphate-binding cations to release P (Kpomblekou and Tabatabai, 1994; Billah et al., 2019). The secretion of organic acids is generally considered to be the main mechanism underlying the mobilization action of lysozyme on insoluble P (Goldstein, 1995; Schefe et al., 2008; Bhattacharyya et al., 2013). The secretion of citric, gluconic, succinic, lactic, and propionic acids by PSBs has been frequently reported in previous studies (Luo et al., 1993; Kim et al., 1997; Chen et al., 2006). These organic acids secreted by PSBs may boost the mobility of insoluble P (mainly chelated with Ca^2+^, Fe^3+^, and Al^3+^) through their hydroxyl and carboxyl groups or by the liberation of protons, thereby converting insoluble P into soluble forms (Kpomblekou and Tabatabai, 1994). In the current study, *Nguyenibacter* sp. L1 inoculated into the growth medium secreted large amounts of gluconic acid relative to other organic acids, and the addition of gluconic acid to the medium also solubilized Al-P. Similarly, gluconic and 2-ketogluconic acids are the main acids implicated in P solubilization in some bacteria (Goldstein, 1995). Our results suggest that *Nguyenibacter* sp. L1 solubilizes Al-P via two mechanisms: by reduction of the pH of the culture medium, and by secretion of gluconic acid. When we decreased the pH of the growth medium to a level equivalent to that obtained by inoculation with *Nguyenibacter* sp. L1, the resulting increase in available P in the culture medium was much lower than that observed following inoculation with *Nguyenibacter* sp. L1. In addition, manual addition of gluconic acid to the culture medium without inoculation with *Nguyenibacter* sp. L1 resulted in more solubilization of Al-P compared with only decreasing the pH of the medium. Secretion of gluconic acid by *Nguyenibacter* sp. L1 also resulted in a decrease in the pH of the culture medium. Secretion of gluconic acid may therefore play a more important role in the solubilization of Al-P by *Nguyenibacter* sp. L1 than reduction of the pH of the culture medium.

According to our study, the secretion of gluconic acid and the solubilization of Al-P by *Nguyenibacter* sp. L1 may depend on the presence of glucose. When fructose was supplied as a carbon source, the amount of dissolved P derived from Al-P and the level of secreted gluconic acid were markedly reduced. Different carbon sources have a great influence on the types and concentrations of organic acids produced by microorganisms and thereby affect P solubility (Relwani et al., 2008). *Aspergillus niger* secretes large amounts of organic acids for dissolving phosphate when starch is used as a carbon source (Li and Qiu, 2011). Compared with fructose, lactose, galactose, and xylose, glucose and sucrose promote significantly higher P solubilization and production of organic acids by *Aspergillus tubingensis* (Relwani et al., 2008). In many bacteria, organic acids are produced from the metabolism of sugars, especially the metabolism of glucose to strong gluconic and 2-ketogluconic acids that solubilize insoluble phosphates (Bashan et al., 2013). Different bacteria use different carbon sources, and, depending on the carbon source, use alternative metabolic pathways to produce different organic acids (Relwani et al., 2008). In the present study, we found that *Nguyenibacter* sp. L1 may use glucose to produce gluconic acid to solubilize Al-P.

*Lespedeza bicolor* is a leguminous shrub that is well adapted to poor acid soil conditions, where P deficiency and Al toxicity often occur (Dong et al., 2008; Sun et al., 2008; Chen et al., 2010). Citrate, oxalate, and malate play important roles in alleviating Al toxicity to plants (Ma et al., 2001). In this study, we found that gluconic acid secreted by *Nguyenibacter* sp. L1 is able to increase the solubilization of Al-P and to alleviate Al toxicity to plants. A previous study has also suggested that *Nguyenibacter* is a N-fixing bacterium (Thi Lan Vu et al., 2013). Plants have evolved various mechanisms to coadapt to multiple stresses such as Al toxicity, low P and high ammonium in acid soil (Zhao et al., 2014; Zhao and Shen, 2018). Taking all of these findings into consideration, we speculate that the colonization of the rhizosphere soil of *L. bicolor* by *Nguyenibacter* sp. L1 contributes to the strong adaptation of this plant to poor acid soils.

## Conclusion

*Nguyenibacter* sp. L1, a new Al-P-solubilizing strain, was isolated from the rhizosphere soil of healthy *L. bicolor* plants growing in acid soil. The solubilization of Al-P by *Nguyenibacter* sp. L1 is associated with the secretion of gluconic acid in the presence of glucose as a carbon source. Gluconic acid can also alleviate Al toxicity to plants.

## Data Availability Statement

The datasets presented in this study can be found in online repositories. The names of the repository/repositories and accession number(s) can be found below: https://www.ncbi.nlm.nih.gov/genbank/, MW774243.

## Author Contributions

XZ and XL conceived, designed the research, and wrote the manuscript draft. XL conducted all of the experiments, analyzed the data, and prepared the figures. XD, JM, and RS revised the manuscript. All authors approved the submitted version.

## Conflict of Interest

The authors declare that the research was conducted in the absence of any commercial or financial relationships that could be construed as a potential conflict of interest.

## Publisher’s Note

All claims expressed in this article are solely those of the authors and do not necessarily represent those of their affiliated organizations, or those of the publisher, the editors and the reviewers. Any product that may be evaluated in this article, or claim that may be made by its manufacturer, is not guaranteed or endorsed by the publisher.
